# PCR-Based Analysis of ColE1 Plasmids in Clinical Isolates and Metagenomic Samples Reveals Their Importance as Gene Capture Platforms

**DOI:** 10.3389/fmicb.2018.00469

**Published:** 2018-03-16

**Authors:** Manuel Ares-Arroyo, Cristina Bernabe-Balas, Alfonso Santos-Lopez, Maria R. Baquero, Kashi N. Prasad, Dolores Cid, Carmen Martin-Espada, Alvaro San Millan, Bruno Gonzalez-Zorn

**Affiliations:** ^1^Departamento de Sanidad Animal and Centro de Vigilancia Sanitaria Veterinaria (VISAVET), Facultad de Veterinaria, Universidad Complutense de Madrid, Madrid, Spain; ^2^Departamento de Microbiología, Facultad de Veterinaria, Universidad Alfonso X el Sabio, Madrid, Spain; ^3^Department of Microbiology, Sanjay Gandhi Postgraduate Institute of Medical Sciences, Lucknow, India; ^4^Servicio de Microbiología Hospital Universitario Ramón y Cajal, Instituto de Investigación Sanitaria (IRYCIS), Madrid, Spain

**Keywords:** antibiotic resistance, ColE1 plasmids, detection PCR, capture PCR, sentinel plasmids

## Abstract

ColE1 plasmids are important vehicles for the spread of antibiotic resistance in the Enterobacteriaceae and Pasteurellaceae families of bacteria. Their monitoring is essential, as they harbor important resistant determinants in humans, animals and the environment. In this work, we have analyzed ColE1 replicons using bioinformatic and experimental approaches. First, we carried out a computational study examining the structure of different ColE1 plasmids deposited in databases. Bioinformatic analysis of these ColE1 replicons revealed a mosaic genetic structure consisting of a host-adapted conserved region responsible for the housekeeping functions of the plasmid, and a variable region encoding a wide variety of genes, including multiple antibiotic resistance determinants. From this exhaustive computational analysis we developed a new PCR-based technique, targeting a specific sequence in the conserved region, for the screening, capture and sequencing of these small plasmids, either specific for Enterobacteriaceae or specific for Pasteurellaceae. To validate this PCR-based system, we tested various collections of isolates from both bacterial families, finding that ColE1 replicons were not only highly prevalent in antibiotic-resistant isolates, but also present in susceptible bacteria. In Pasteurellaceae, ColE1 plasmids carried almost exclusively antibiotic resistance genes. In Enterobacteriaceae, these plasmids encoded a large range of traits, including not only antibiotic resistance determinants, but also a wide variety of genes, showing the huge genetic plasticity of these small replicons. Finally, we also used a metagenomic approach in order to validate this technique, performing this PCR system using total DNA extractions from fecal samples from poultry, turkeys, pigs and humans. Using Illumina sequencing of the PCR products we identified a great diversity of genes encoded by ColE1 replicons, including different antibiotic resistance determinants, supporting the previous results achieved with the collections of bacterial isolates. In addition, we detected cryptic ColE1 plasmids in both families with no known genes in their variable region, which we have named sentinel plasmids. In conclusion, in this work we present a useful genetic tool for the detection and analysis of ColE1 plasmids, and confirm their important role in the dissemination of antibiotic resistance, especially in the Pasteurellaceae family of bacteria.

## Introduction

Plasmids are autonomously replicating fragments of extra-chromosomal DNA that can be transferred horizontally between bacteria. They usually harbor genes that confer a selective advantage under adverse conditions, becoming a major source of genetic variability in bacteria and playing a key role in their adaptation and evolution (Baquero, 2011; Wiedenbeck and Cohan, 2011). Antimicrobial resistance has become one of the most serious problems in public health, and the concern about the ability of plasmids to spread antimicrobial resistance determinants has greatly increased (Carattoli, 2013).

ColE1-type plasmids (hereafter ColE1 plasmids) are small, mobilizable, multi-copy replicons, found mainly in Enterobacteriaceae and Pasteurellaceae (Tomizawa et al., 1977; San Millan et al., 2007), although they have been described in other families of bacteria (Pan et al., 2010; Vincent et al., 2016). These plasmids have a distinctive theta replication mechanism, regulated by two small RNAs encoded close to the origin of replication (*oriV*) (Tomizawa, 1984; Lilly and Camps, 2015). According to the sequences of their mobilization genes, ColE1 replicons belong to the MOB_P_ family of plasmids (Garcillán-Barcia et al., 2009, 2015).

Several works have shown that ColE1 plasmids are carriers of resistance mechanisms of high clinical relevance. In Enterobacteriaceae, these plasmids are present in commensal (Pallecchi et al., 2010, 2011; Anantham and Hall, 2012; Moran and Hall, 2017) and pathogenic isolates (de Toro et al., 2013; Garbari et al., 2015; Stoesser et al., 2016, 2017; Albornoz et al., 2017), where they have been found to carry genes conferring resistance to fluoroquinolones, aminoglycosides, sulfonamides and β-lactams, including antibiotics of last resort such as carbapenems (Papagiannitsis et al., 2015) and even colistin (Borowiak et al., 2017). In Pasteurellaceae, several ColE1 plasmids conferring resistance to tetracyclines, aminoglycosides, sulfonamides and β-lactams have been described in human and animal pathogens of the genera *Pasteurella* (San Millan et al., 2009), *Haemophilus* (San Millan et al., 2010, 2011; Tristram et al., 2010; Moleres et al., 2015) and *Actinobacillus* (Blanco et al., 2007). Furthermore, in Pasteurellaceae species of animal origin, ColE1 plasmids are probably the main vehicle for the acquisition of antibiotic resistance determinants (Lancashire et al., 2005; Blanco et al., 2006, 2007; San Millan et al., 2007), being postulated as key strategy for multidrug resistance in this family (San Millan et al., 2009).

In this study, we carried out a computational analysis of the genetic structure of ColE1 plasmids in Enterobacteriaceae and Pasteurellaceae. With this data, we developed a novel PCR-based strategy for the detection, capture and study of ColE1 plasmids, and validated this technique using antibiotic susceptible and resistant isolates from Enterobacteriaceae and Pasteurellaceae. The results presented here highlight the importance of ColE1 plasmids as a source of antibiotic resistance, providing a useful tool for the study of these small replicons.

## Materials and methods

### Bacterial strains, sample collection and culture conditions

In this study we used a total number of 135 Pasteurellaceae and 50 Enterobacteriaceae isolates. A collection of 44 *Pasteurella multocida* and 39 *Mannheimia haemolytica* isolates were obtained from the lungs of 3-month-old lambs and are described in Supplementary Table 1. These strains were isolated, identified on the basis of phenotype, this identification being confirmed by species-specific PCR, and then further characterized by serotyping (Fraser et al., 1983; Townsend et al., 1998, 2001; Angen et al., 2002). In addition, 52 Pasteurellaceae isolates from dogs and cats were recovered during 2009–2010, from oral samples cultured on Columbia 5% sheep blood agar plus 16 mg/l bacitracin, and characterized by Gram staining, oxidase tests and a lack of growth on MacConkey agar (BioMérieux, France). They were further characterized with the API 20NE microorganism identification test kit (BioMérieux, France) and by species-specific PCR and sequencing of the 16S rRNA gene (Król et al., 2011). *Haemophilus influenzae* and *Haemophilus parasuis* were cultured on chocolate agar PolyViteX plates (BioMérieux, France) and in *Haemophilus* Test Medium (HTM) broth (Wider, Francisco Soria Melguizo, S.A., Spain) at 37°C in microaerophilic conditions (5% CO_2_) for 48 h. Moreover, 50 multidrug-resistant Enterobacteriaceae isolates were obtained from the Sanjay Gandhi Postgraduate Institute of Medical Sciences, in Lucknow, India. All the strains were isolated in 2010 and their antibiotic resistance and plasmid profiles are described in Supplementary Table 2. The remaining Enterobacteriaceae species and Pasteurellaceae species were cultured on Columbia agar +5% sheep blood plates and in BHI broth (BioMérieux, France) at 37°C for 24 h.

Additionally, we collected fecal samples from different origins: poultry, turkey, pig and human. Animal fecal samples were collected from three different farms located in the center area of Spain during 2015-2016. Each farm produced a different animal species: poultry, turkey and pig, respectively. Twenty-five individual samples of feces were taken from each animal species in each farm, and then pooled into one unique sample, stored at −80° until DNA extraction. Additionally, a fecal sample of a human from the same region was also collected during the same period and stored at the same temperature.

### Antibiotic susceptibility testing

Antimicrobial susceptibility was determined by disk diffusion and microdilution methods, in accordance with CLSI guidelines (CLSI, 2013a,b). Commercially prepared dehydrated Sensititre panels (Trek Diagnostics, Inc., Westlake, OH) were used for MIC determination. Quality control of the panels was performed according to the manufacturer's instructions. Antibiotic disks were obtained from BioMérieux (BioMérieux, France) and Oxoid (Oxoid Ltd., Basingstoke, United Kingdom). Antibiotics were supplied by Merck (Merck KGaA, Darmstadt, Germany) and Sigma-Aldrich (Sigma Chemical Co. St Louis, Mo, USA).

### Computational DNA analysis

**Figure 1:**
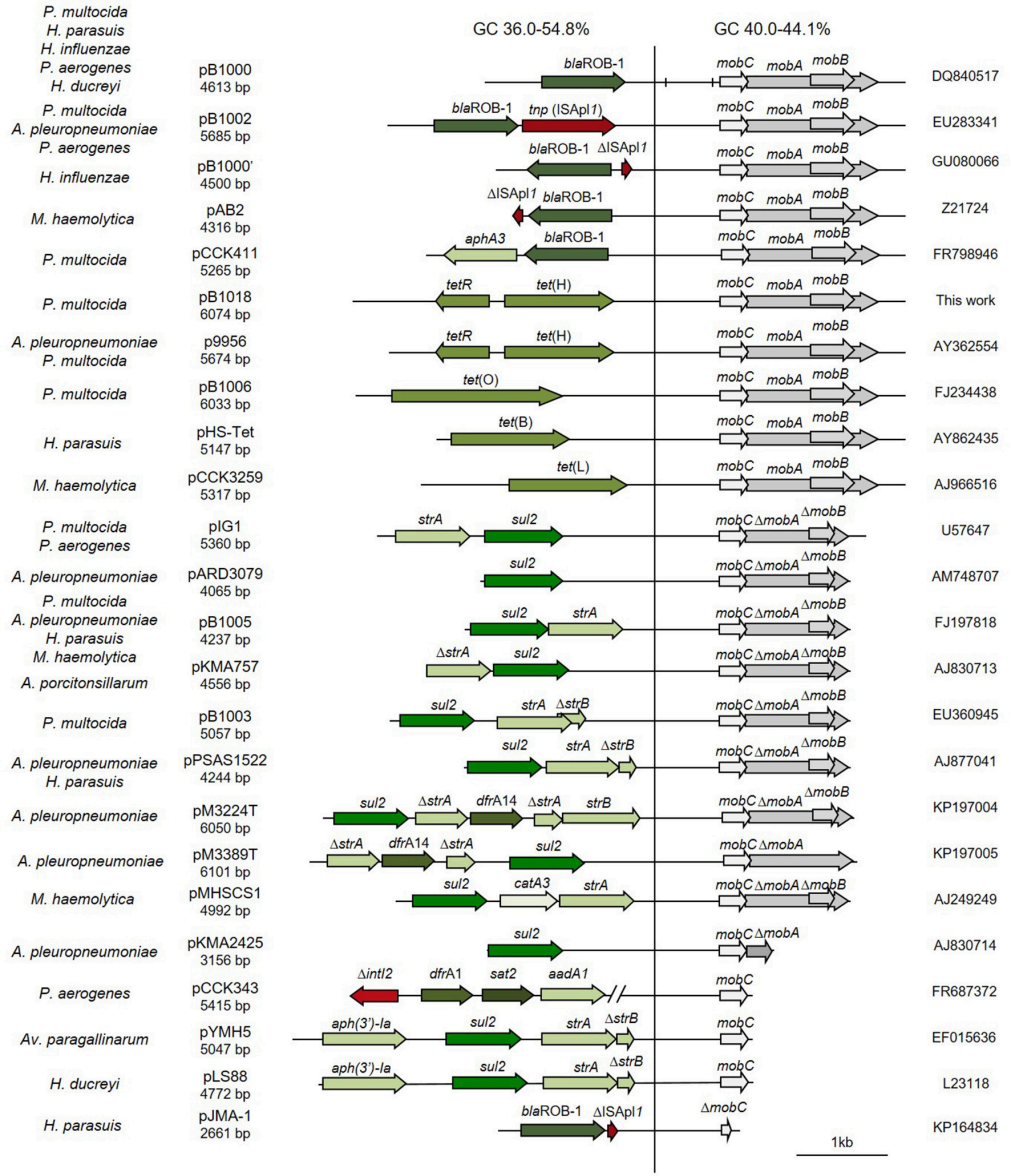
Genetic structure of ColE1 plasmids from Pasteurellaceae. Schematic diagram of ColE1 plasmids from the Pasteurellaceae family. The reading frames for genes are shown as arrows, with the direction of transcription indicated by the arrowhead. Antimicrobial drug resistance determinants are shown in green whereas genes involved in genetic transposition or integration are shown in red. Genes encoding plasmid relaxases are shown in gray. In pB1000, two vertical bars bracket the region containing the putative origin of replication (*oriV*) and the putative origin of transfer (*oriT*). The large vertical bar separates the conserved region of the plasmid, to the right, from the variable region of the plasmid, to the left. Percentage ranges of GC content of variable and conserved regions of the plasmids are indicated in the top of the figure. The species in which the plasmid has been described and the name, size and accession number of plasmids are also indicated.

**Figure 2:**
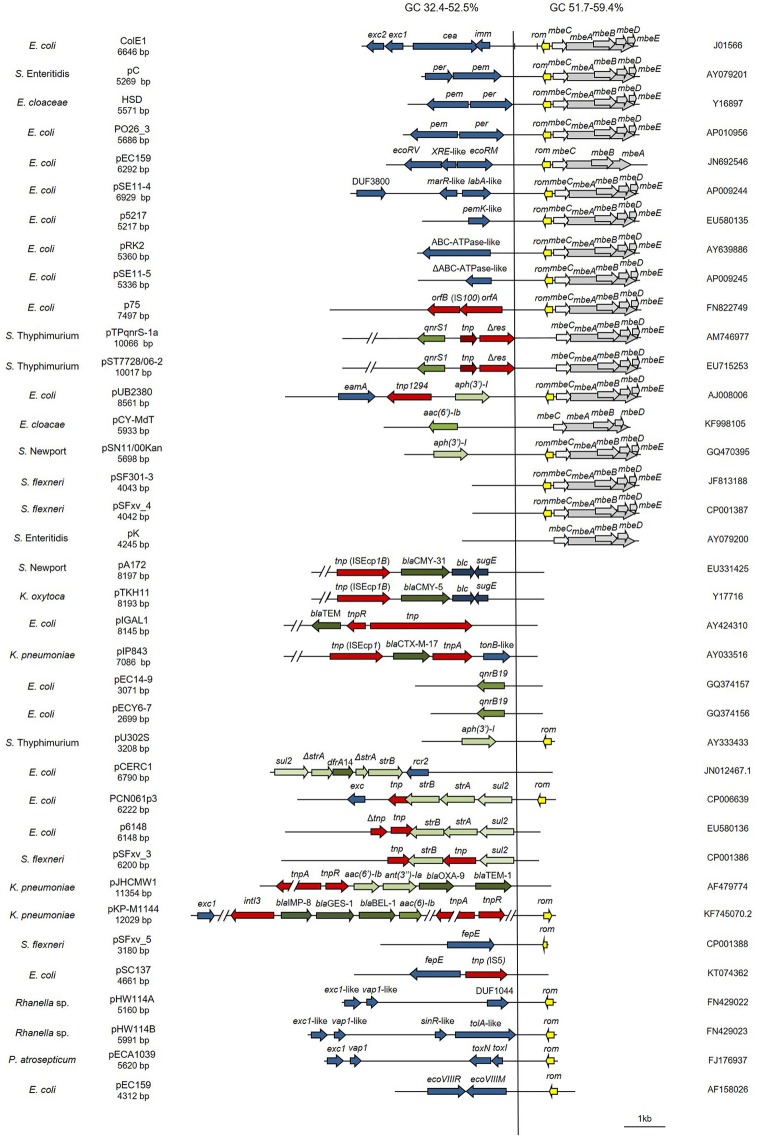
Genetic structure of ColE1 plasmids from Enterobacteriaceae. Schematic diagram of the 37 ColE1 plasmids from the Enterobacteriaceae family studied in this work. The reading frames for genes are shown as arrows, with the direction of transcription indicated by the arrowhead. The names of the genes, or the names of the family of proteins they encode, are indicated. Antimicrobial drug resistance genes are shown in green and genes involved in genetic transposition or integration are shown in red. Genes encoding plasmid relaxases are shown in gray and the rom gene implicated in the regulation of plasmid replication is shown in yellow. The remaining ORFs are shown in blue. In ColE1, two vertical bars bracket the region containing the origin of replication (*oriV*) and the origin of transfer (*oriT*). The large vertical bar separates the conserved region of the plasmids, to the right, from the variable region of the plasmids, to the left. Percentage ranges of GC content of variable **(Left)** and conserved **(Right)** regions of the plasmids are indicated in the top of the figure. The species in which the plasmid has been described, and the name, size, and accession number of plasmids are also indicated.

Data on complete plasmid sequences were obtained from GenBank. The structure of ColE1 from Pasteurellaceae was studied in the complete sequence of 24 wild type plasmids (Figure 1). The structure of ColE1 from Enterobacteriaceae was studied in the complete sequence of 37 wild type plasmids (Figure 2), excluding the numerous sequences from cloning vectors or other ColE1-based genetic tools. For the recovery of wild type ColE1 plasmids, short input sequence BLAST of different 20 bp segments including the origin of replication of ColE1 plasmids was performed (http://blast.ncbi.nlm.nih.gov/). The BLASTs were performed excluding all ColE1-derived cloning vectors, and only ColE1 plasmids from wild type strains were analyzed. The conserved region of plasmids was determined independently in Pasteurellaceae and Enterobacteriaceae by sequence alignments using Megablast. Plasmid sequence identity score of plasmid conserved regions was established using ColE1 (Accession no. J01566) and pB1000 (Accession no. DQ840517) as prototypes for ColE1 plasmids from Enterobacteriaceae and Pasteurellaceae families, respectively. Nucleotide BLASTs of the conserved region were performed to establish sequence identity using Nblast and Megablast. Conserved region of plasmid ColE1 was established from nucleotide 641 to 3,940, from the start of RNAII to the end of *mbeE* gene. The conserved region of plasmid pB1000 was established from nucleotide 3,584 to 2,665, excluding *bla*_ROB−1_ gene. Highly related sequences were selected to determine the GC content (%) in the conserved region using Serial Cloner 2.1 (Serial Basics, France). The regions with no homology were used to estimate Guanine plus Cytosine percentage in the variable region of the plasmids. Differences between variances were tested with the F test to compare the variances of two samples from normal populations. Different software were used for sequence analysis: 4Peaks 1.6 (Mek&Tosj, Netherlands) for the analysis of chromatograms, NIH online analysis tools (http://www.ncbi.nlm.nih.gov) for DNA alignments, and DNA Strider 1.4f13 (CEA, France) and CLC DNA workbench (CLC bio, Cambridge, MA) for general features like full integration of the data or analysis of the annotated sequences.

### *In vitro* DNA analysis

The plasmids used in this study are shown in Figures 1, 2, **4**, **6**. Plasmid DNA from bacteria isolates was extracted with Plasmid Midi and QIAprep Spin Miniprep (Qiagen, Inc., Chatworth, California, USA). PCR products were purified with Qiagen PCR Purification or Gel Extraction kits (Qiagen, Inc., Chatworth, California, USA). PCR was performed with Taq polymerase from Biotools (B&M Labs, Spain), Phusion high-fidelity DNA polymerase (Finnzymes, Woburn, MA, USA), AmpliTaq Gold DNA polymerase (Applied Biosystems, AB, Foster City, CA, USA) and Taq-Core (Qbiogene, Carlsbad, CA, USA). Automated Sanger sequencing of six complete plasmids was carried out with an Abi-Prism Apparatus (Perkin-Elmer) at Secugen S. L. (Madrid, Spain). The *P. multocida* isolates were identified by PCR detection of the *kmt* gene with the species-specific primers KMT1T7 and KMT1SP6 (Townsend et al., 1998). Capsular type was determined by the PCR method described by Townsend et al. (2001).

### Detection PCR for ColE1 plasmids

DNA-free polymerases, such as AmpliTaq Gold DNA polymerase (Applied Biosystems, Foster City, CA, USA) and Taq-Core (Qbiogene, Carlsbad, CA, USA), are required for this test in Enterobacteriaceae species to avoid false positive results (Supplementary Figure 1). Crude bacterial lysate can act as DNA template for this reaction. Detection PCR was performed according to the kit manufacturer's instructions, with primers ColE1 detF (tgaacggggggttcgtgca)/ColE1 detR (cgtttttccataggctccgcc) for Enterobacteriaceae, producing a PCR product of about 300 bp; and ColE1-P detF (gtctccgtttcgtgctacggt)/ColE1-P detR (aaatcagcggagccgataggc) for Pasteurellaceae, producing a PCR product of about 450 bp. The amplification conditions were: initial denaturation for 5 min at 94°C, followed by 25 cycles of denaturation for 30 s at 94°C, annealing for 30 s at 55°C and extension for 30 s at 72°C, with a final extension phase for 10 min at 72°C.

### Capture PCR for ColE1 plasmids

**Figure 3:**
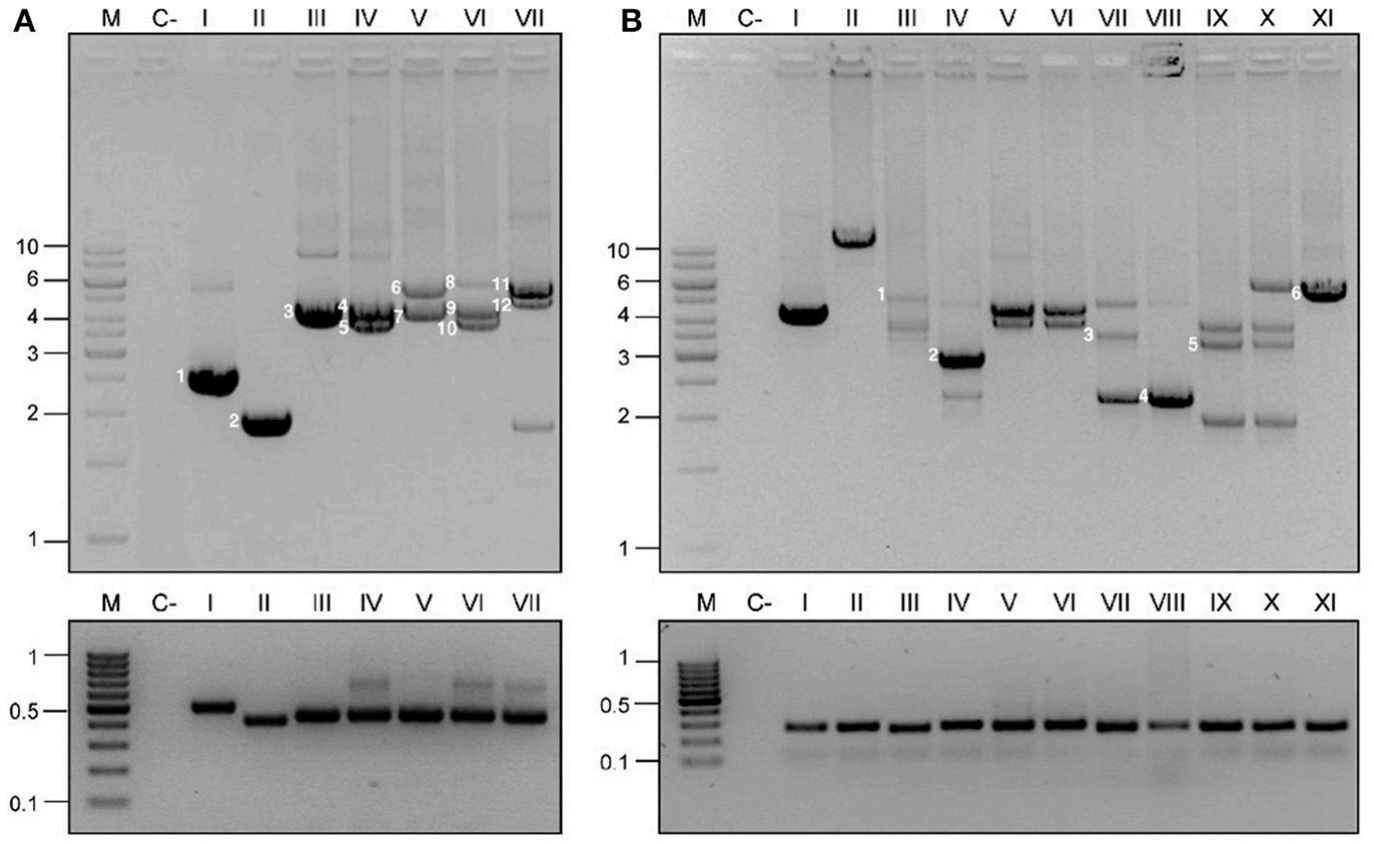
Detection and capture PCR for ColE1 plasmids. ColE1 detection (bottom) and capture (top) PCRs in Pasteurellaceae **(A)** and Enterobacteriaceae **(B)**. M stands for molecular weight marker and numbers to the left of the panels indicate the size in kb. C- Stands for negative control. The lanes correspond to different strains and are indicated with roman numbers. **(A)** Pasteurellaceae family PCRs. Negative control (c-) corresponds to *H. influenzae* RdKW20. The products of the capture PCR, corresponding to the ColE1 plasmids carried by the strains, are indicated by numbers. Lane I, *P. stomatis* BB1086: pB000a (1). Lane II, *Frederiksenia canicola* BB1087: pB000b (2). Lane III, *H. influenzae* BB1059: pB1000 (3). Lane IV, *P. multocida* BB1035: pB1000 (4) and pB1005 (5). Lane V, *P. multocida* BB1041: p9956 (6) and pB1000 (7). Lane VI, *P. multocida* BB1044: pB1000 (9), pB1005 (10) and pB1006 (8). Lane VII, *P. multocida* BB1046: pB1002 (11) and pB1003 (12). **(B)** Enterobacteriaceae family PCRs. Negative control (c-) represents *E. coli* DH5α. Lanes I and II correspond to ColE1 based cloning vectors pTOPO and pUC19 (with the insertion of a ~13 kb DNA fragment), respectively. Lanes III to XI correspond to wild type strains from the Sanjay Gandhi Postgraduate Institute of Medical Sciences in India. Lanes III-VII, *K. pneumonia*. Lane VIII, *P. mirabilis*. Lanes IX and X, *E. cloacae*. Lane XI, *E. coli*. Six random plasmids were completely sequenced from these strains, and are indicated by numbers in the agarose gel: pB1019 (1), pB1020 (2), pB1022 (4), pB1023 (5), and pB1024 (6).

Capture PCR was performed with the Phusion high-fidelity DNA polymerase (Finnzymes, Woburn, MA, USA). No false positive results were observed using this polymerase for the capture PCR. This PCR has been shown to amplify pUC19 vectors with insertions generating products of up to 15 kb (Figure 3). Plasmid preparations using kits as the QIAprep Spin Miniprep (Qiagen, Inc., Chatworth, California, USA) are the recommended DNA template. PCR was performed with the ColE1 cap-1 (tgcacgaaccccccgttca)/ColE1 cap-2 (ggcggagcctatggaaaaacg) primers for Enterobacteriaceae and the ColE1-P cap-1 (accgtagcacgaaacggagac)/ColE1-P cap-2 (gcctatcggctccgctgattt) primers for Pasteurellaceae, according to the kit manufacturer's instructions. The following conditions were used: initial denaturation for 30 s at 98°C, followed by 30 cycles of denaturation at 98°C for 10 s, annealing at 56°C for 10 s and extension at 72°C for 4 min, with a final extension phase for 10 min at 72°C.

### Sequencing and analysis of metagenomic samples

We extracted the total DNA from the four fecal samples with QIAmp Fast DNA Stool Mini Kit (Qiagen, Inc., Chatworth, California, USA), and then performed the Capture PCR from this total DNA. After purifying the amplified DNA with the Qiagen PCR Purification kit (Qiagen, Inc., Chatworth, California, USA), it was sequenced and assembled at MicrobesNG (Birmingham, United Kingdom) by Next Generation Sequencing following their standard analysis pipeline.

DNA was quantified in triplicates with the Quantit dsDNA HS assay in an Ependorff AF2200 plate reader. Genomic DNA libraries were prepared using Nextera XT Library Prep Kit (Illumina, San Diego, USA) following the manufacturer's protocol with two modifications: two nanograms of DNA instead of one were used as input, and PCR elongation time was increased to 1 min from 30 s. DNA quantification and library preparation were carried out on a Hamilton Microlab STAR automated liquid handling system. Pooled libraries were quantified using the KapaBiosystems Library Quantification Kit for Illumina on a Roche light cycler 96 qPCR machine. Libraries were sequenced on the Illumina MiSeq using a 250 bp paired end protocol. Reads were adapter trimmed using Trimmomatic 0.30 with a sliding window quality cutoff of Q15 (Bolger et al., 2014). *De novo* assembly was performed on samples using SPAdes version 3.7 (Bankevich et al., 2012). Additional information about the data of this sequencing is presented in Supplementary Table 3.

An automated annotation of the contigs assembled was performed at MicrobesNG using Prokka (Seemann, 2014). Moreover, we developed an additional annotation of the sequences combining the analysis tools RAST (Aziz et al., 2008) and ResFinder (Zankari et al., 2012). In order to assure that the annotations were actually present in ColE1 replicons, we analyzed the genetic environment of the genes in the contigs assembled at MicrobesNG. We assumed that these genes were encoded actually on ColE1 replicons when their whole contig sequence upstream and downstream the gene corresponded to ColE1-like sequences according to the NIH online analysis tool Nucleotide BLAST (http://blast.ncbi.nlm.nih.gov/).

### Nucleotide sequence accession numbers

Nucleotide sequences of the plasmids obtained in this study have been deposited in GenBank under the following accession numbers: pB1018 from BB1253, JQ319774; pB000a from BB1086, JQ319773; pB000b from BB1087, JQ319771; pB1019 from BB1088, JQ319775; pB1020 from BB1089, JQ319772; pB1021 from BB1090, JQ319767; pB1022 from BB1091, JQ319766; pB1023 from BB1092, JQ319770; and pB1024 from BB1093, JQ319768.

## Results

### Computational analysis of ColE1 plasmids

#### ColE1 plasmids in pasteurellaceae

The bioinformatic analysis of the ColE1 plasmids from Pasteurellaceae species led to the identification of two differentiated genetic regions (Figure 1): a conserved region carrying all the elements controlling replication and transfer, and a variable region encoding mainly antibiotic resistance genes. The conserved region of ColE1 plasmids from Pasteurellaceae had an average size of 2,513 bp (Standard Deviation, *SD* = 718 bp) and was highly similar among all of the plasmids, with a GC content between 40.0 and 44.1% and high nucleotide sequence identity (average = 97.62%, SD = 2.06%) (Figure 1). In contrast, the variable region of these ColE1 plasmids presented a high level of genetic divergence. As result of this variability, the GC content in this region varied between 36.0 and 54.8%. The variance of the GC content was significantly lower in the conserved region than in the variable region [*F*_(23)_ = 40.22, *P* < 0.001].

#### ColE1 plasmids in enterobacteriaceae

In this family, the ColE1 replicons also showed a conserved area involved in plasmid housekeeping functions and a variable region. However, in contrast to plasmids from Pasteurellaceae, ColE1 plasmids in Enterobacteriaceae encoded a wide variety of accessory genes, including also antibiotic resistance determinants (Figure 2). The conserved region of ColE1 plasmids from Enterobacteriaceae had an average size of 1,817 bp (*SD* = 1,197 bp) with an average nucleotide sequence identity of 82.59% (*SD* = 10.79%). This region presented a GC content between 51.7 and 59.4%, whereas in the variable region GC content varied between 32.4 and 52.5%. Again, the variance of the GC content was significantly lower in the conserved region [*F*_(36)_ = 14.11, *P* < 0.001].

### Development of a PCR-based system for detection and capture of ColE1 plasmids

We used the data obtained from the *in silico* analysis of the sequences of wild type ColE1 plasmids to develop a two PCR-based system for the detection and capture of ColE1 replicons in Enterobacteriaceae and Pasteurellaceae. Pairwise and multiple DNA alignments of the conserved sequences of the plasmids were performed in order to detect conserved regions suitable for the design of PCR primers (for detailed description of the PCRs conditions and primers see Material and Methods).

As the nucleotide sequence of ColE1 plasmids presents a highly conserved region among all the replicons belonging to the same bacterial family (82.59% in Enterobacteriaceae and 97.62% in Pasteurellaceae), but drastically different when compared against plasmids from the other family, we were forced to design two different set of primers, each one specific for Enterobacteriaceae ColE1 plasmids and Pasteurellaceae ColE1 plasmids, respectively.

Thus, we developed a two PCRs system for the specific analysis of ColE1 plasmids in each family of bacteria. First, a “Detection PCR” using a pair of universal primers was designed to amplify a small fragment from the conserved region of the replicons, close to the *oriV*. Second, we designed a “Capture PCR,” using a pair of primers annealing to the exact same region as the primers form the detection PCR, but amplifying outwards, allowing the capture of the variable region of the plasmid. Using this technique, the whole plasmid sequence is available for further analysis.

### Validation of the PCR-based system for ColE1 analysis

#### Validation in ColE1 plasmids from pasteurellaceae

In order to validate the PCR-based system in Pasteurellaceae, we used a well-characterized series of strains of *H. influenzae* (San Millan et al., 2010, 2011), *H. parasuis* (San Millan et al., 2007) and *P. multocida* (San Millan et al., 2009; Santos-Lopez et al., 2017). These strains, previously described by our group, carried one, two or three ColE1 plasmids. The Detection PCR was positive in every isolate and, most importantly, the Capture PCR was not only able to amplify a single ColE1 plasmid, but it gave rise to different PCR products, corresponding to each coexisting plasmid in those strains carrying multiple (up to three) plasmids. As negative controls we used the reference strains *H. influenzae* RdKW20, *P. multocida* ATCC 43137 and *H. parasuis* ATCC 19417, which do not carry ColE1 plasmids. As expected, no PCR product was observed in any of these strains. In Figure 3A we show the results of a representative group of Pasteurellaceae strains tested: *H. influenzae* RdKW20, *P. stomatis* BB1086, *Frederiksenia canicola* BB1087, *H. influenzae* BB1059, *P. multocida* BB1035, *P. multocida* BB1041, *P. multocida* BB1044, *P. multocida* BB1046.

**Figure 4:**
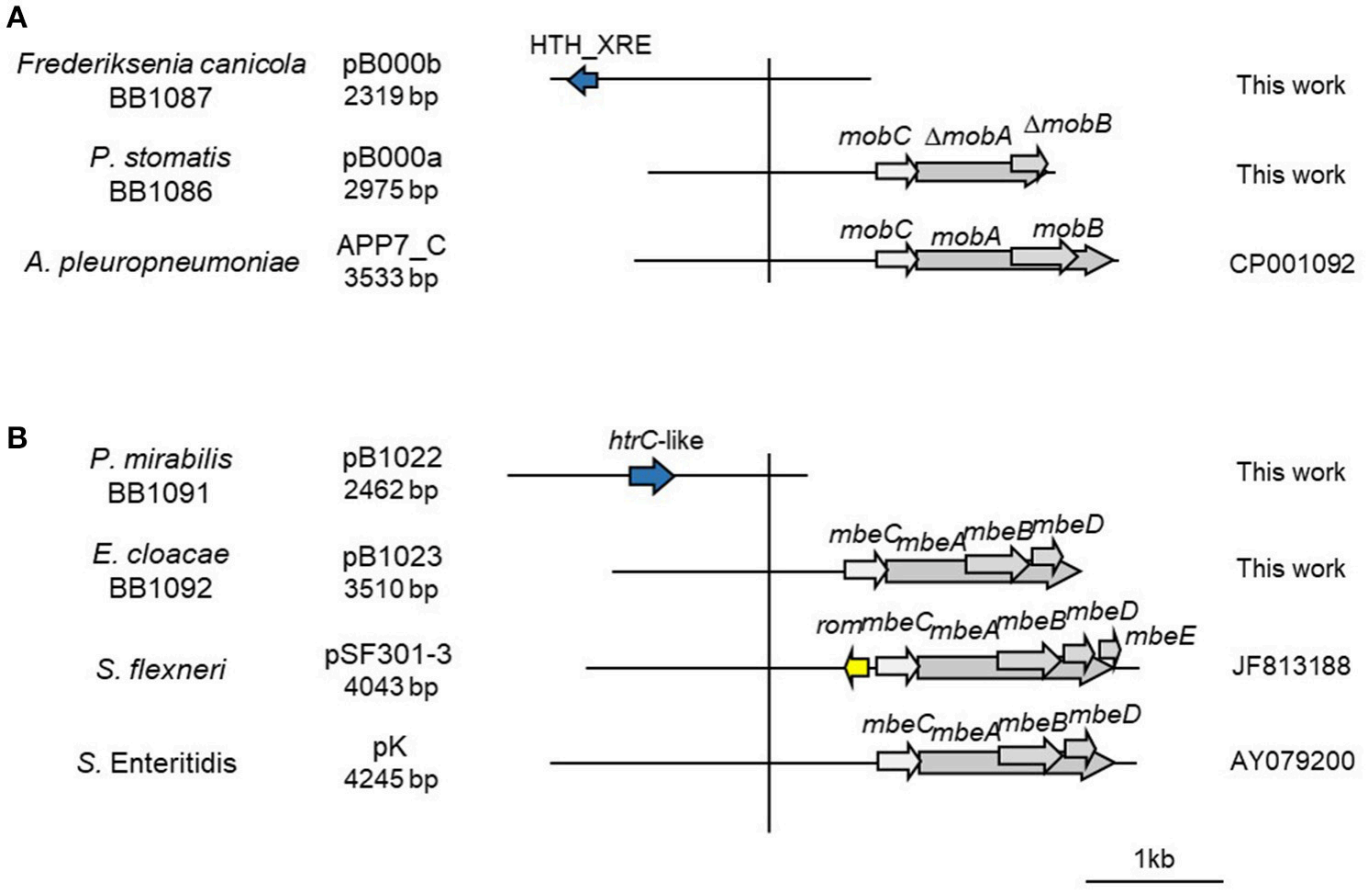
Genetic structure of a selection of ColE1 cryptic plasmids. Schematic diagram of ColE1 cryptic plasmids from Pasteurellaceae **(A)** and Enterobacteriaceae **(B)**. The reading frames for genes are shown as arrows, with the direction of transcription indicated by the arrowhead. Genes encoding plasmid relaxases are shown in gray. The vertical bar separates the conserved region of the plasmids, to the right, from the variable region of the plasmids, to the left. The species in which the plasmid has been described, and the name, size, and accession number of plasmids are also indicated.

Additionally, we decided to establish two new collections of Pasteurellaceae isolates to test the PCR-based system in a wider range of species. We constructed a first collection of 52 Pasteurellaceae isolates from oral samples collected from healthy dogs and cats, including strains from six different Pasteurellaceae species: *P. multocida, Pasteurella canis, Pasteurella pneumotropica, Pasteurella stomatis, Pasteurella dagmatis* and *F. canicola*. A second collection was collected from lungs of 3-month-old lambs counting with 44 *P. multocida* and 39 *M. haemolytica* strains (Supplementary Table 1). We performed antibiotic susceptibility testing for clinically relevant antibiotics in both collections, detecting eight tetracycline resistant *P. multocida* strains in the lamb collection (Supplementary Table 1). We carried out the ColE1 Detection PCR and detected 10 positive strains; the eight tetracycline resistant *P*. *multocida* isolates from the lamb collection and two susceptible isolates from the dog collection: a *P. stomatis* (BB1086) and a *F. canicola* (BB1087). The Capture PCR was also positive in these 10 strains. In the eight tetracycline resistant *P. multocida* the Capture PCR revealed the presence of a plasmid of about 6 kb in size in all the isolates. This plasmid, bearing the tetracycline resistance gene *tet(H)*, was completely sequenced and named pB1018 (Figure 1). The strains from the dog collection harbored two plasmids of 2,975 bp (BB1086) and a 2,319 bp (BB1087) in size, respectively. These cryptic plasmids carried no detectable known gene in their variable region, and we named them pB000a and pB000b (Figure 4A).

#### Validation in ColE1 plasmids from enterobacteriaceae

We first tested the PCR system using ColE1-based cloning vectors, such as pTOPO and pUC19. The Detection PCR was positive and the Capture PCR was able to amplify fragments of up to 15 kb in size from genetic constructions using the ColE1-based pUC19 plasmid (Figure 3B). Therefore, this reaction should be able to capture any ColE1 plasmid from wild type strains. As negative controls we used laboratorial strains carrying no ColE1 plasmids as *Escherichia coli* DH5α. In contrast to the case of Pasteurellaceae, we did not have access to a previously characterized collection of Enterobacteriaceae strains carrying ColE1 plasmids. Hence, we decided to analyse a new collection of 50 clinical isolates of Enterobacteriaceae, including six different species and displaying resistance to various antibiotics, recovered at the Sanjay Gandhi Postgraduate Institute of Medical Sciences in India (Supplementary Table 2). Thirty seven of fifty isolates gave positive results in the ColE1 Detection PCR (Figure 3B). The amplicons from a representative number of strains were sequenced, and were confirmed to have originated from ColE1 plasmids. Thus, 74% of the Enterobacteriaceae studied isolates actually carried at least one ColE1 plasmid. The Capture PCR was then performed for the isolates bearing ColE1 plasmids, and it generated from one to three amplicons per isolate, with sizes ranging from 2 to 10 kb, corresponding to the various ColE1 plasmids present in the cell (Figure 3B). This result was confirmed by partial sequencing of the different PCR products after DNA purification from agarose gel (see Material and Methods).

**Figure 5:**
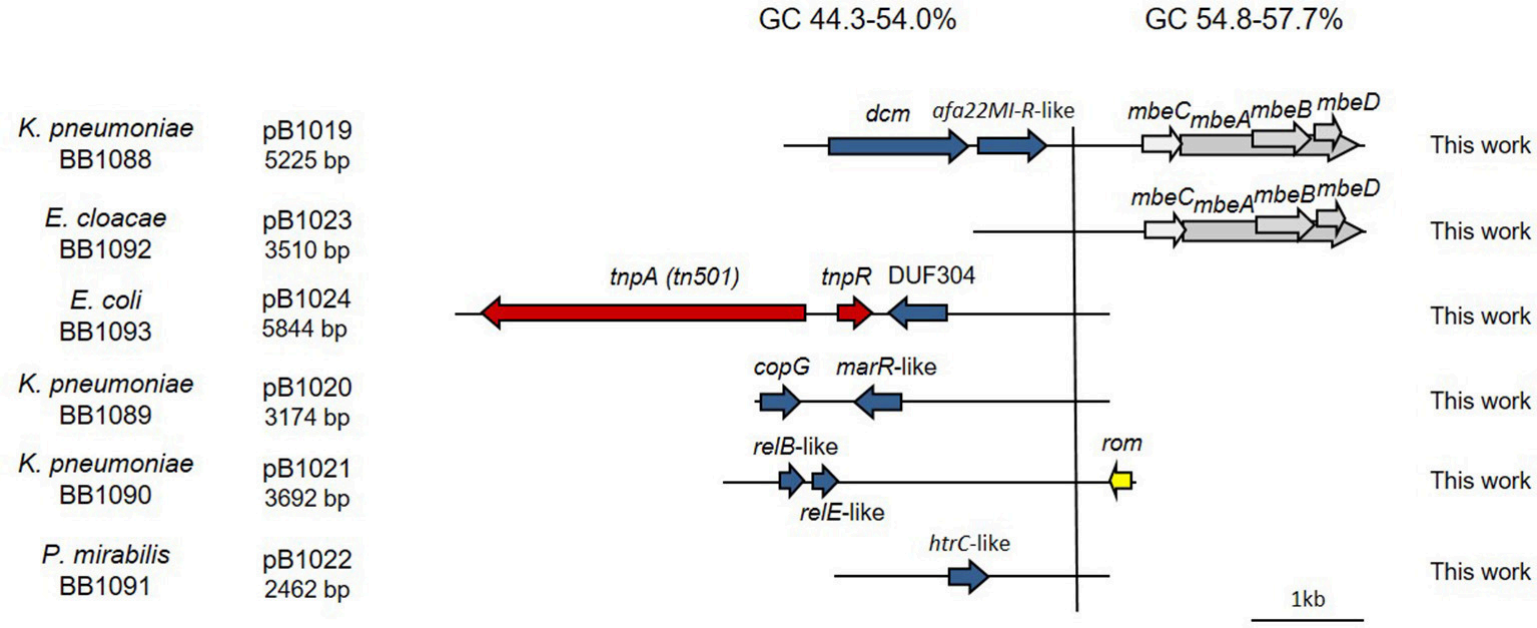
Genetic structure of ColE1 plasmids from Enterobacteriaceae clinical isolates. Schematic diagram of the ColE1 plasmids from the Enterobacteriaceae family described here. The reading frames for genes are shown as arrows, with the direction of transcription indicated by the arrowhead. The names of the genes, or the names of the family of proteins they encode, are indicated. Genes involved in genetic transposition or integration are shown in red. Genes encoding plasmid relaxases are shown in gray and the *rom* gene involved in the regulation of plasmid replication is shown in yellow. The remaining ORFs are shown in blue. Percentage ranges of GC content of variable and conserved regions of the plasmids are indicated in the top of the figure. The vertical bar separates the conserved region of the plasmids, to the right, from the variable region of the plasmids, to the left. The strain in which the plasmid was described, and the name and size of the plasmids, are also indicated.

We completely sequenced six random ColE1 plasmids (pB1019 to pB1024) from this collection of strains to confirm the results and the utility of the method developed here (Figure 5). These sequenced replicons had the typical characteristics of ColE1 plasmids, with both the conserved and the variable regions. Again, the GC content of the conserved region was very similar among these plasmids (54.8–57.7%), whereas the plasmid variable region presented diverse GC content (44.3–54.0%). We found different genes in the variable region of these replicons: toxin-antitoxin systems (pB1021), restriction-modification systems (pB1019) or transposases like Tn501 (pB1024). Interestingly, we also found two cryptic plasmids (pB1022 and pB1023). However, no antibiotic resistance gene was found in these plasmids despite the high level of resistance of the strains.

It is important to mention that contamination with DNA from ColE1 cloning vectors in some of the commercial DNA polymerases generated a false-positive reaction in the ColE1 Detection PCR in Enterobacteriaceae (Supplementary Figure 1). DNA-free polymerases, such as AmpliTaq Gold DNA polymerase (Applied Biosystems) and Taq-Core (Qbiogene), should therefore be used for this PCR reaction. Such contamination, leading to erroneous PCR results, has been described before and is of particular relevance in the case of the *bla*_TEM−1_ and *bla*_TEM−116_ β-lactamases genes (Koncan et al., 2007; Jacoby and Bush, 2016).

#### Validation in ColE1 plasmids from intestinal microbiota

In order to validate if the PCR system is useful for the analysis of ColE1 plasmids from metagenomic samples, we decided to test the Capture PCR directly on total DNA extracted from fecal samples using the primers specific for ColE1 plasmids from Enterobacteriaceae. Four pools of fecal samples were tested in this study, three of them collected from healthy animals of different species: poultry, turkey and pig; and a fourth one from human origin. We did not use the PCR-system for Pasteurellaceae given the limited presence of these bacteria in the gut microbiota of both animals and humans (Roto et al., 2015; Burrough et al., 2017; Gupta et al., 2017). After performing the Capture PCR and purifying the final product, we sequenced the amplified DNA resulting from all the ColE1 plasmids harbored in the Enterobacteriaceae cells present in the fecal samples using Illumina MiSeq. We analyzed the genes present on the amplicons, and their genetic environment, confirming that they were actually present in ColE1 plasmids. We found several mobilization genes, transposases, toxin-antitoxin systems, bacteriocins and restriction-modification systems (Table 1), in addition to several hypothetical proteins ranging between 32 and 418 amino acids. Interestingly we also observed multiple antibiotic resistance genes conferring resistance to some of the most important antibiotic families such as ß-lactams, aminoglycosides and fluoroquinolones (Table 1).

**Table 1:** List of genes harbored by ColE1 plasmids in the fecal samples.

**NAME**	**DESCRIPTION**	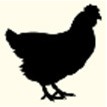	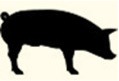	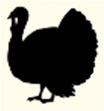	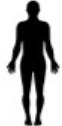
**REPLICATION**					
*rom*	RNAI modulator protein	^*^	^*^	^*^	^*^
**CONJUGATION**					
*mbeA*	Mobilization protein	^*^	^*^	^*^	^*^
*mbeB*-like	Mobilization protein	^*^			
*mbeC*	Mobilization protein	^*^	^*^	^*^	^*^
*mbeD*	Mobilization protein	^*^			
*virB4*	Type IV secretion system protein		^*^		^*^
**ANTIBIOTIC RESISTANCE**					
*aph(3′)-IIa*	Aminoglycoside phosphotransferase	^*^	^*^		^*^
*bla*_TEM−116_	β-Lactamase	^*^	^*^	^*^	^*^
*strA*	Aminoglycoside phosphotransferase		^*^	^*^	
*strB*	Aminoglycoside phosphotransferase		^*^		
*sul2*	Sulfonamide-resistance dihydropteroate synthase		^*^		
*qnrB19*	Quinolone resistance protein			^*^	^*^
**TRANSPOSASES**					
IS91	Transposase		^*^		
IS5	Transposase			^*^	^*^
IS3	Transposase				^*^
Tn3	Transposon				^*^
**TOXIN-ANTITOXIN SYSTEMS**					
*ccdB*	CcdB/CcdA Toxin protein	^*^			^*^
*higA*	HigB/HigA Antitoxin protein	^*^		^*^	^*^
*higB*	HigB/HigA Toxin protein				^*^
*lsoA*	LsoA/LsoB Toxin protein				^*^
*parE*	ParE/ParD Toxin protein	^*^		^*^	
*pemI*	PemK/PemI Antitoxin protein				^*^
*pemK*	PemK/PemI Toxin protein				^*^
**BACTERIOCINS**					
*caa*	Colicin-A	^*^	^*^		
*cai*	Colicin-A immunity protein		^*^		
*cea*	Colicin-E1	^*^	^*^		
*imm*	Colicin-E1 immunity protein				^*^
*lys*	Colicin-E1 lysis protein				^*^
*csa*	Colicin-S4			^*^	
*csi*	Colicin-S4 immunity protein			^*^	
*cta*	Colicin-10		^*^	^*^	^*^
*kba*	Klebicin-B				^*^
*kbi*	Klebicin-B immunity protein				^*^
**RESTRICTION-MODIFICATION SYSTEMS**					
*ecoRVR*	Restriction endonuclease EcoRV	^*^	^*^	^*^	
*ecoRVM*	DNA-methyltransferase EcoRV	^*^			
*ecoVIIIR*	Restriction endonuclease EcoVIII			^*^	
*ecoVIIIM*	DNA-methyltransferase EcoVIII			^*^	
*banIR*	Restriction endonuclease BanI	^*^		^*^	^*^
*banIM*	DNA-methyltransferase BanI				^*^
*styD4IR*	Restriction endonuclease StyD4I	^*^			^*^
*styD4IM*	DNA-methyltransferase StyD4I	^*^			^*^
*eco29kIR*	Restriction endonuclease Eco29kI	^*^			
*eco29kIM*	DNA-methyltransferase Eco29kI	^*^			
*eco034IR*	Restriction Enzyme Eco034I	^*^			^*^
*eco034IM*	DNA-methyltransferase Eco034I	^*^			
*ydiOR*	Restriction endonuclease YdiO		^*^		
*ydiOM*	DNA-methyltransferase YdiO		^*^		
**MISCELLANEOUS**					
*abi*-like	Abortive infection bacteriophage protein		^*^	^*^	^*^
*chrB*	Membrane chromate resistance protein				^*^
*copG* family	Ribbon-helix-helix protein	^*^	^*^	^*^	
*csp*	Cold shock protein	^*^		^*^	^*^
*era*	GTPase Era			^*^	
*exc1*	Entry exclusion protein 1	^*^	^*^	^*^	^*^
*exc2*	Entry exclusion protein 2	^*^			^*^
*escC* family	Type III secretion system protein	^*^		^*^	^*^
*fepE*	Ferric enterobactin transport protein	^*^	^*^	^*^	^*^
Helix-turn-helix	Helix-turn-helix domain containing protein	^*^	^*^	^*^	^*^
*htrC*	Heat shock protein C	^*^	^*^	^*^	^*^
*kdgF*	Pectin degradation protein				^*^
*nicB*	Nicotine dehydrogenase subunit B	^*^			
*nikA*	Nickel ABC transporter substrate-binding protein	^*^	^*^	^*^	^*^
*parA*	Plasmid partitioning protein		^*^		
Phage integrase	Phage integrase family	^*^			
Phosphatase 2C	Type 2C protein phosphatase			^*^	^*^
*pspF*	Phage shock protein operon transcriptional activator			^*^	^*^
Reverse transcriptase motif	RNA-dependant DNA-polymerase	^*^		^*^	^*^
*rihC*	Ribonucleoside hydrolase	^*^			
*tsr*	Methyl-accepting chemotaxis protein		^*^		^*^
*xre*	Transcriptional regulator XRE family protein			^*^	
*ydaM*	Diguanylate cyclase, csgD regulator		^*^		
**DIMER RESOLUTION**					
xerD	Site-specific tyrosine recombinase	^*^			^*^
xerC	Site-specific tyrosine recombinase	^*^			^*^

The name and description of the genes is given on the left side of the table, while the asterisks (^*^) of the right side indicate the presence of each gene in the different samples, representing from left to the right: poultry, pig, turkey and human.

## Discussion

**Figure 6:**
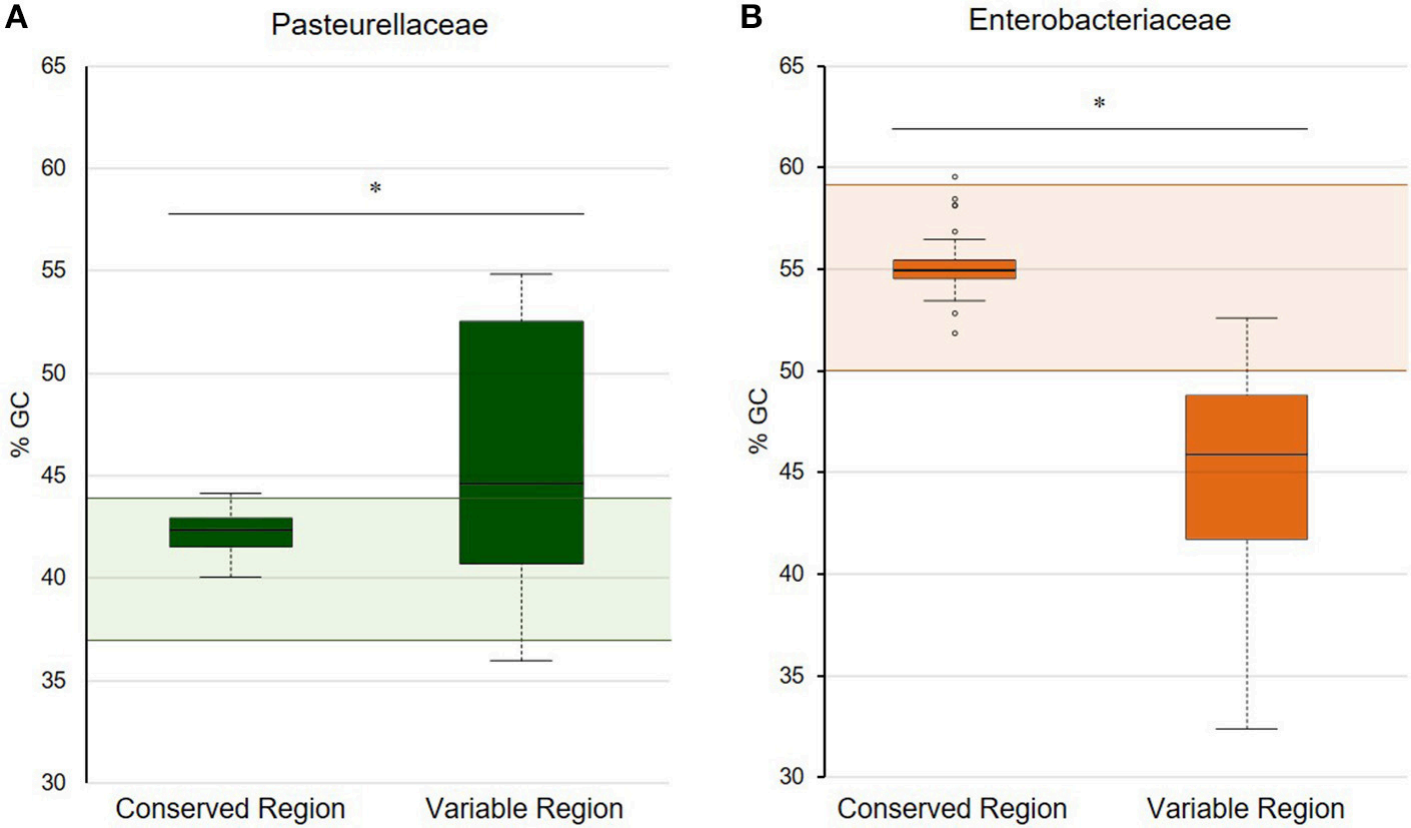
GC content of the conserved and variable regions of ColE1 plasmids. Representation of the percentage of GC content in both the conserved and variable region of ColE1 plasmids in Pasteurellaceae **(A)** and Enterobacteriaceae **(B)**. Colored boxplots represent the GC content of each of the ColE1 replicons studied. The shaded areas correspond to the %GC range of the genomes analyzed of these two bacteria families (Supplementary Tables S1, S2). The asterisks indicate the difference of the mean values of GC content between the conserved and variable region in both families of bacteria [*t*_(24)_ = 2.91, *P* < 0.05 and *t*_(41)_ = 10.83, *P* < 0.05, respectively].

In this work, we analyzed the structure and content of ColE1 plasmids described in Pasteurellaceae and Enterobacteriaceae up to date. Our results showed that although ColE1 plasmids are different in these two families, they presented the same genetic structure. We observed two differentiated regions in the plasmids, one highly conserved and another one highly variable. The conserved region harbored all the housekeeping functions of the plasmid, including the origins of replication (*oriV*) and transfer (*oriT*) and, in most of plasmids analyzed, relaxases genes. The GC content of this conserved region is similar to the GC content of the genomes in which ColE1 plasmids have been found (Figure 6): 37–44% in Pasteurellaceae (Supplementary Table 3) and 50–59% in Enterobacteriaceae (Supplementary Table 4), strongly suggesting a plasmid/host adaptation process. On the other hand, the variable region carries accessory genes from different origins. In this variable region we found a wide variety of antibiotic resistance determinants in both families. In Pasteurellaceae, we found genes conferring resistance to tetracycline, β-lactams, aminoglycosides, sulphonamides, trimethoprim and chloramphenicol (Figure 1). In contrast to the plasmids from Pasteurellaceae, ColE1 plasmids from Enterobacteriaceae carried a wide variety of genes apart from antibiotic resistance determinants: genes involved in resistance to phage infections such as abortive infection systems (Fineran et al., 2009) and restriction-modification systems (Gregorova et al., 2002), genes involved in ferric transport (Ye et al., 2010) or genes encoding bacteriocins, such as the colicin E1 (Tomizawa et al., 1977). However, despite the higher qualitative diversity in the genes encoded in their variable region, ColE1 plasmids from Enterobacteriaceae carried antibiotic resistance genes, mainly against β-lactams, aminoglycosides and sulphonamides (Figure 2).

Using the data obtained from our computational analysis, we developed a new PCR-based system able to detect and completely capture ColE1 plasmids in Enterobacteriaceae and Pasteurellaceae. This is, to the best of our knowledge, the first system of this nature developed for ColE1 plasmids in Pasteurellaceae. In Enterobacteriaceae, previous PCR-based tests (García-Fernández et al., 2009; Chen et al., 2010; Alvarado et al., 2012) have been described for the detection of ColE1 plasmids.

García-Fernández et al. (2009) designed a set of primers targeting a conserved region in the origin of replication of ColE1 plasmids while looking for plasmids harboring quinolone resistance genes in *Salmonella*. With these primers (Table 2) they successfully detected three different ColE1 replicons, demonstrating the efficacy of this PCR. However, by testing these primers *in silico* against the ColE1 represented in Figure 2, just 14 out of the 37 plasmids carried the complete sequence for primers hybridization. In addition, Chen et al. (2010) developed a PCR-based system for the detection of ColE1 plasmids in *Salmonella*, by using primers targeting a conserved region within the origin of replication and the *rom* gene (Table 2). However, we also tested these primers *in silico* and just 13 out of the 37 replicons did harbor the whole sequence complementary to these oligonucleotides. In parallel to the previous techniques targeting the origin of replication, Alvarado et al. (2012) developed a Degenerate Primer MOB Typing (DPMT) technique, extremely useful to detect and classify plasmids present in gamma-proteobacteria by targeting their relaxases genes. This DPMT included different degenerate primers against the MOB_P5_ relaxases of ColE1 plasmids (Table 2). However, as these genes are actually absent in a substantial proportion of ColE1 replicons (Figure 2), a considerably part of these plasmids would not be detected by using only this set of primers. In summary, our bioinformatic analysis revealed that these prior methods, although scrupulously designed and useful in the particular studies in which they were employed, would fail to detect part of the wild type ColE1 plasmids described to date, either for lack of sensitivity of the primers or for targeting the mobilization genes. Nevertheless, in order to reach the most sensitivity as possible, we suggest the combination of all these primers to assure the detection of any ColE1 plasmid present in a sample.

**Table 2:** Primers used to detect ColE1 plasmids in Enterobacteriaceae isolates in previous studies.

**Primer name**	**Sequence (5′-3′)**	**Length**	**Reference**
oricolE Fw	GTTCGTGCATACAGTCCA	18	García-Fernández et al., 2009
oricolE Rv	GGCGAAACCCGACAGGACT	19	
CC7059F	TTCGTGCACACAGCCCA	17	Chen et al., 2010
CC7062R	TGCGGTTATCCACAGAATCA	20	
CC7063F	GCGGACAGGTATCCGGTAA	19	
P51-f	TACCACGCCCTATGCGAARAARTAYAC	27	Alvarado et al., 2012
P52-f	GATAGCCTTGATTTTAATAACACCAAYACYTAYAC	35	
P5-r	CCCTTGTCCTGGTGYTSNACCCA	23	
P53-f	GGGCTCGCACGAYCAYACNGG	21	
P53-r	GCCCAGCCCTTTTCRTGRTTRTG	23	
ColE1 detF	TGAACGGGGGGTTCGTGCA	19	This work
ColE1 detR	CGTTTTTCCATAGGCTCCGCC	21	

In order to validate our technique, we used the PCR system in a range of bacterial collections from Enterobacteriaceae and Pasteurellaceae as well as in metagenomic samples from fecal origin. These experiments revealed interesting results. In Pasteurellaceae we confirmed the tight link between ColE1 plasmids and antibiotic resistance. Moreover, we discovered the presence of cryptic ColE1 plasmids in antibiotic susceptible *P. stomatis* BB1086 (pB000a) and *F. canicola* BB1087 (pB000b), which only encoded plasmid housekeeping genes. In Enterobacteriaceae, the PCR-based screening system showed that the prevalence of the ColE1 replicons in the antibiotic resistance collection tested was especially high, with the 74% of the isolates carrying at least one plasmid. We also detected cryptic ColE1 plasmids (pB1022 and pB1023) with no evident genes in their variable region in this collection (Figure 4B). Other cryptic ColE1 plasmids, such as pB000a, pB000b, pB1022, and pB1023, have been previously described in human, animal and environmental isolates (Rozhon et al., 2006; Handford et al., 2009; Bleicher et al., 2013) (Figure 4) and, interestingly, some of the antibiotic resistance genes encoded in ColE1 replicons had been described in different bacterial genus and families (Miranda et al., 2003; Soge et al., 2006; Warburton et al., 2016). Hence, our hypothesis is that these unexpected prevalent cryptic replicons might act as “sentinel plasmids,” capable of maintaining just the conserved region in bacteria due to their capacity of replication and conjugation (Burian et al., 1997), but able to acquire a wide variety of genes from heterogeneous origins, providing an increased genetic plasticity to their host.

In addition, our metagenomic approach confirmed the large diversity of genes that these small replicons can encode in the gut enterobacteria in healthy animals and humans (Table 1). We consider important to mention that we do not suggest a species distribution of the ColE1 genes based on our sample collection. However, we kept it separated in Table 1, firstly to show that there is no bias in our approach, and secondly, as it could be interesting in future works aiming to study the epidemiology of ColE1 plasmids. Many of the detected genes have well known functions in plasmids biology, as mobilization genes, toxin-antitoxin systems (Moran and Hall, 2017), the previously cited restriction-modification systems or the bacteriocins. However, the function of other genes found in this sample, and previously described in other ColE1 plasmids such as the *copG*-like genes (de Toro et al., 2013), are still unknown. Of especial relevance in these ColE1 amplicons from fecal samples was the detection of antibiotic resistance determinants against quinolones, aminoglycosides, sulphonamides and β-lactams. Previous works showed that the most prevalent antibiotic resistance genes in human and animal gut microbiomes are those conferring resistance to tetracycline, representing even the 90% of the gut resistome (Durso et al., 2012; Pal et al., 2016). However, although ColE1 plasmids frequently carry tetracycline resistance genes in Pasteurellaceae (Figure 1), they are not common in Enterobacteriaceae (Figure 2) and none has been detected in our approach. Other resistance genes are less represented in animal and human microbiomes, although new techniques with higher sensitivity (Lanza et al., 2018) might highlight the presence of genes that have been underrepresented to date in metagenomics samples. In our study, the genes detected with our ColE1 capture PCR were the *qnrB19* quinolone resistance gene in turkey and human, the *aph(3*′*)-IIa* aminoglycoside phosphotranspherases in poultry, pig and human, or the *strA* gene in turkey, which was found both alone and forming a *strA-strB-sul2* complex in the pig sample, being all these genes previously described in ColE1 replicons (Figure 2). The only antibiotic resistance determinant found in all the species was the *bla*_TEM−116_ gene, which encodes an extended-spectrum β-lactamase (ESBL) (Lahlaoui et al., 2011). TEM-116 has been recently described as the central node of one of the two TEM clusters that group all the known TEM variants (Zeil et al., 2016), emphasizing its importance in antimicrobial resistance evolution and the role of ColE1 plasmids in its dissemination.

In conclusion, we have developed a simple system to screen and characterize ColE1 plasmids, which will allow monitoring the increasingly relevant role of these plasmids in the spread and evolution of antibiotic resistance in Pasteurellaceae and Enterobacteriaceae.

## Author contributions

MA-A, CB-B, and AS-L have contributed with the design of the work, data collection, analysis and interpretation, drafting the article, and AS-L witch critical revision of the draft. MB, CM-E, DC, and KP have contributed with strains and critical revision of the article for final approval. AS has contributed with the design of the work, data collection and analysis, and critical revision of the draft. BG-Z has contributed with the conception and design of the work, interpretation of the data collection and analysis, critical revision of the draft and final approval of the article.

### Conflict of interest statement

The authors declare that the research was conducted in the absence of any commercial or financial relationships that could be construed as a potential conflict of interest.
