# Supplementary material for: PCR-Based Analysis of ColE1 Plasmids in Clinical Isolates and Metagenomic Samples Reveals Their Importance as Gene Capture Platforms
Source: Front Microbiol. 2018 Mar 16;9:469. doi: 10.3389/fmicb.2018.00469 (PMC5864857; doi:10.3389/fmicb.2018.00469)
Supplement: Supplementary file 1 [file DataSheet1.docx]

Supplementary Material

**PCR-based analysis of ColE1 plasmids in clinical isolates and metagenomic samples reveals their importance as gene capture platforms.**

**Manuel ARES-ARROYO, Cristina BERNABE-BALAS, Alfonso SANTOS-LOPEZ, Maria Rosario BAQUERO, Kashi N. PRASAD, Dolores CID, Carmen MARTIN-ESPADA, Alvaro SAN MILLAN and Bruno GONZALEZ-ZORN***

*** Correspondence:** Bruno GONZALEZ-ZORN: bgzorn@ucm.es

# Supplementary Figure


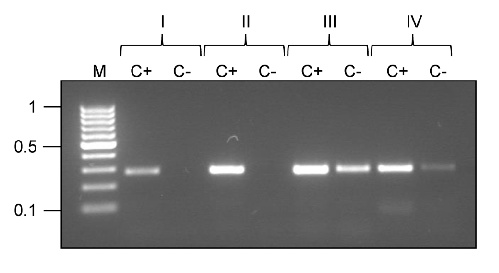


**Supplementary Figure S1**. False-positive reaction in ColE1 detection PCR in Enterobacteriaceae.

Contamination with DNA from ColE1 in some of the commercial DNA polymerases tested generated a false-positive reaction in ColE1 detection PCR in Enterobacteriaceae. Detection PCR for ColE1 plasmids from Enterobacteriaceae using different commercially DNA polymerases: I, AmpliTaqe Gold DNA polymerase (Applied Biosystems, AB, Foster City, CA, USA). II, Taq-Core (Qbiogene, Carlsbad, CA, USA). III, Taq polymerase from Biotools (B&M Labs, Spain). IV, Phusion high-fidelity DNA polymerases (Finnzymes, Woburn, MA, USA). C+ and C- stand for positive (pUC19) and negative (water) control, respectively.

# Supplementary Tables

**Supplementary Table 1.** *Pasteurella multocida and Mannheimia haemolytica* isolated from lamb lung samples.

| **Strain** | | | **Resistance phenotype^a^** | **ColE1-P plasmids** | Serotype^b^ | Isolation year |
| --- | --- | --- | --- | --- | --- | --- |
|  | | |  |  |  |  |
| *Pasteurella multocida* | | |  |  |  |  |
| P91 P104 P114 P127 P141 | P94 P109 P116 P130 | P103 P112 P117 P140 | S | - | A | 2008 |
| P92 P99 P110 P139 P145 | P93 P107 P118 P142 | P97 P108 P132 P144 | S | - | D | 2008 |
| P61 P85 | P63 | P72 | S | - | A | 2009 |
| P60 | P65 |  | S | - | D | 2009 |
| P147 | P148 |  | S | - | ND | 2009 |
| BB1253 P76 P84 | P67 P80 | P75 P83 | Tet | + | A | 2009 |
| P77 | | | Tet | + | ND | 2009 |
|  | | |  |  |  |  |
| *Mannheimia haemolytica* | | |  |  |  |  |
| M1 | M14 |  | S | - | A1 | 2008 |
| M12 M32 | M26 M36 | M29 | S | - | A2 | 2008 |
| M58 M81 | M59 M220 | M65 | S | - | A5 | 2008 |
| M141 M169 M210 | M158 M196 | M162 M205 | S | - | A6 | 2008 |
| M28 | M30 |  | S | - | A7 | 2008 |
| M180 | | | S | - | A8 | 2008 |
| M88 | M110 | M173 | S | - | A9 | 2008 |
| M5 M50 M103 M130 M234 | M9 M52 M106 M144 M237 | M48 M55 M115 M182 | S | - | ND | 2008 |

a, Antibiotics tested: amoxicillin, amoxicillin-clavulanate, ceftiofur, enrofloxacin, florfenicol, gentamycin, penicillin, streptomycin, and tetracycline (TET). S, susceptible to every antibiotic tested.

b, Serotyping was performed as previously described for *P. multocida* (Townsed *et al.*, 2001), and *M. haemolytica* (Fraser *et al.*, 1983). ND, not determined by these methods.

**Supplementary Table 2.** Clinical isolates of Enterobacteriaceae from India.

| Strain | | | | Resistance phenotype^a^ | ColE1 plasmids |
| --- | --- | --- | --- | --- | --- |
|  | | | |  |  |
| *Escherichia coli* | | | |  |  |
| I1 | I7 | I29 | I36 | CFS, FEP, CAZ, Ci, CF, G, TzP | + |
| I4 | I30 | I35 | | FEP, CAZ, Ci, CF, G, TzP | + |
| I5 | I28 | | | CAZ, Ci, CF, G, TzP | + |
| I15 | | | | FEP, CAZ, Ci, G, TzP | - |
| I33 | | | | CAZ, Ci, Nx | + |
| I50 | | | | Caz, Ci, Cf | - |
| I116 | | | | CFS, Caz, Ci, Cf, Ert, G, Mem, Tzp | - |
| I119 | | | | Caz, Ci, Cf, G | - |
| BB1093 | | | | Caz, Ci, G, Le | + |
| I137 | | | | Caz, Ci, G | + |
|  | | | |  |  |
| *Klebsiella pneumoniae* | | | |  |  |
| BB1088 | | | | FEP, CAZ, Ci, CF, G, TzP | + |
| I3 I20 | I10 I22 | I11 I24 | I13 | CFS, FEP, CAZ, Ci, CF, G, TzP | + |
| I16 | I17 | I31 | I32 | CFS, FEP, CAZ, Ci, CF, G, TzP | - |
| I19 | | | | CAZ, Ci, G, Nf, Nx | + |
| I23 | | | | CAZ, Ci, CF, Tzp | + |
| I27 | | | | FEP, CAZ, Ci, CF, G, TzP | + |
| I37 | | | | CAZ, Ci | + |
| I38 | | | | CFS, CAZ, Ci, G, Nx | + |
| I39 | | | | Cfs, Caz, Ci, Cf, TZP | + |
| BB1089 | | I42 | | Cfs, fep, Caz, Ci, Cf, TZP | + |
| I43 | | | | fep, Caz, Ci, Cf, Mem | + |
| I145 | | | | Cfs, Caz, Ci, Cf, Ert, G, TZP, Tig | - |
| I149 | | | | Cfs, Caz, Ci, Cf, G, Tig | - |
| BB1090 | | | | Cfs, fep, Caz, Ci, Cf, Mem, TZP | + |
|  | | | |  |  |
| *Citrobacter freundii* | | | |  |  |
| I9 | | | | CFS, CAZ, Ci, G | + |
| I26 | | | | CFS, FEP, CAZ, Ci, CF, G, TzP | + |
| I49 | | | | Caz, Ci, Cf, TZP | + |
| I146 | | | | Cfs, Caz, Ci, Cf, G | - |
|  | | | |  |  |
| *Proteus mirabilis* | | | |  |  |
| I46 | | | | CfS, CaZ, Ci, TZP | - |
| BB1091 | | | | Cfs, CaZ, Ci, Cf | + |
|  | | | |  |  |
| *Enterobacter cloacae* | | | |  |  |
| BB1092 | | I81 | | Cfs, CaZ, Ci, Ert, G, TZP | + |
|  | |  | |  |  |
| *Enterobacter aerogenes* | | | |  |  |
| I21 | | | | CFS, CAZ, Ci, G, Nf, Nx | + |
| I69 | | | | Cfs, Caz, Ci, Cf, TZP | - |

a, Antibiotics tested: cefoperazone (CFS), cefepime (FEP), ceftazidime (CAZ), ceftriaxone (CI), ciprofloxacin (CF), colistin (CT), cotrimoxazol (CO), ertapenem (ERT), gentamicin (G), imipenem (IMP), levofloxacin (LE), meropenem (MEM), nitrofurantoin (NF), norfloxacin (NX), pipercillin tazobactam (TZP), and tigecycline (TIG).

**Supplementary Table 3.** Summary of the statistics of the reads and assemblies of the sequencing data.

| Sample | Median Insert Size | Mean Coverage | Number of Reads | Number of Reads with Insert Size > 300 | Number of Contigs (>= 0 bp) | Number of Contigs (>=1000bp) | Total Length (>=0bp) | Total Length (>=1000bp) | Largest Contig | Total Length | GC (%) |
| --- | --- | --- | --- | --- | --- | --- | --- | --- | --- | --- | --- |
| 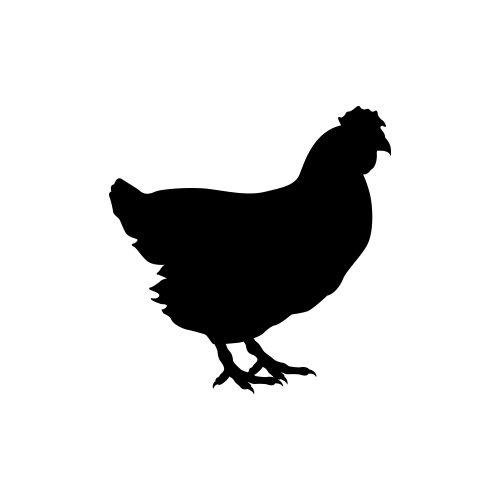 | 412 | 529.464 | 1446163 | 556939 | 3839 | 86 | 1153287 | 194194 | 7435 | 345283 | 49.31 |
| 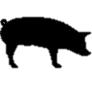 | 348 | 615.414 | 1123059 | 365672 | 2781 | 32 | 767346 | 84242 | 6955 | 139651 | 47.73 |
| 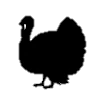 | 467 | 553.632 | 434729 | 192355 | 1075 | 43 | 341749 | 101317 | 6882 | 130358 | 45.52 |
| 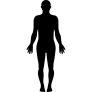 | 382 | 159.244 | 1029653 | 302962 | 10996 | 70 | 2712984 | 145439 | 6389 | 391774 | 47.84 |

**Supplementary Table 4.** GC content in a representative number of Pasteurellaceae species.

| Species | Genome GC% | Number of genomes | | Accession number(s) |
| --- | --- | --- | --- | --- |
|  |  | |  |  |
| *Actinobacillus minor* | 39 | | 2 | NZ_ACQL00000000, NZ_ACFT00000000 |
| *Actinobacillus pleuopneumoniae* | 40-41 | | 15 | NC_009053, NC_010939, NC_010278, NZ_ADXN00000000, NZ_ADXO00000000, NZ_AACK00000000, NZ_ADOM00000000, NZ_ADOL00000000, NZ_ADOK00000000, NZ_ADOJ00000000, NZ_ADOI00000000, NZ_ADOG00000000, NZ_ADOF00000000, NZ_ADOE00000000, NZ_ADOD00000000 |
| *Actinobacillus succinogenes* | 44 | | 1 | NC_009655 |
| *Actinobacillus ureae* | 39 | | 1 | NZ_AEVG00000000 |
| *Aggregatibacter actinomycetemcomitans* | 44 | | 14 | NC_017846, NC_013416, NC_016513, ADOB00000000, ADOA00000000, AHGR00000000, AEJK00000000, AEJQ00000000, AEJP00000000, AEJR00000000, AEJL00000000, AEJM00000000, AEJN00000000, AEJO00000000 |
| *Aggregatibacter aphrophilus* | 42 | | 3 | NC_012913, AEWB00000000, ACZJ00000000 |
| *Aggregatibacter segnis* | 42 | | 1 | NZ_AEPS00000000 |
| *Avibacterium paragallinarum* | 40 | | 1 | NZ_AFFP00000000 |
| *Gallibacterium anatis* | 39 | | 1 | NC_015460 |
| *Haemophilus aegypticus* | 38 | | 1 | NZ_AFBC00000000 |
| *Haemophilus ducreyi* | 38 | | 1 | NC_002940 |
| *Haemophilus haemolyticus* | 38 | | 6 | NZ_AFQQ00000000, AJSV00000000, AFQN00000000, AFQO00000000, AFQP00000000, AFQR00000000 |
| *Haemophilus influenzae* | 37-38 | | 20 | NC_000907, NC_014922, NC_009566, NC_009567, NC_007146, NC_014920, NC_016809, NC_017452, NC_017451, NZ_ABWV00000000, NZ_ABWW00000000, NZ_AAZJ00000000, NZ_AAZH00000000, NZ_AAZG00000000, NZ_AAZF00000000, NZ_AAZE00000000, NZ_AAZD00000000, NZ_AAZI00000000, NZ_ACSL00000000, NZ_ACSM00000000 |
| *Haemophilus parainfluenzae* | 39 | | 4 | NC_015964, NZ_AEWU00000000, AJTC00000000, AJMW00000000 |
| *Haemophilus parasuis* | 39 | | 2 | NC_011852, NZ_ABKM00000000 |
| *Haemophilus pittmaniae* | 42 | | 1 | NZ_ABKM00000000 |
| *Histophilus somni* | 37 | | 2 | NC_010519, NC_008309 |
| *Mannheimia haemolytica* | 40-41 | | 3 | NZ_ACZX00000000, NZ_ACZY00000000, NZ_AASA00000000 |
| *Mannheimia succiniciproducens* | 42 | | 1 | NC_006300 |
| *Pasteurella dagmatis* | 37 | | 1 | NZ_ACZR00000000 |
| *Pasteurella multocida* | 40 | | 6 | NC_002663, NC_016808.1, NC_017764, NC_017027, AFRR00000000, AFRS00000000 |

**Supplementary Table 5.** GC content in a representative number of Enterobacteriaceae species.

| Species | Genome GC% | Number of genomes | | Accession number(s) |
| --- | --- | --- | --- | --- |
|  |  | |  |  |
| *Cronobacter dublinensis* | 58 | | 1 | NZ_CP012266.1 |
| *Edwarsiella sp.* | 59 | | 2 | NZ_CP011364.1, NZ_CP011516.1 |
| *Enterobacter cloacae* | 55-56 | | 7 | NZ_CP012162.1, NZ_CP017990.1, CP016906.1, FP929040.1, NZ_LT160614.1, NZ_LT840187.1, NZ_CP014280.1 |
| *Escherichia coli* | 50-51 | | 20 | NC_002695.1, NC_011750.1, NC_000913.3, NC_017634.1, NC_018658.1, CU928163.2, NC_012892.2, :NC_007779.1, NC_011415.1, NC_013654.1, NC_012947.1, NC_017628.1, NC_011748.1, NC_011741.1, NC_017651.1, NC_017663.1, NC_017638.1, NC_020163.1, NZ_HG738867.1, NZ_CP007392.1 |
| *Klebsiella michiganensis* | 55-56 | | 3 | NC_016612.1, NZ_CP004887.1, NZ_CP011077.1 |
| *Klebsiella oxytoca* | 55-56 | | 8 | NZ_CP011636.1, NZ_CP008788.1, NZ_CP011618.1, NZ_CP011597.1, NZ_AP014951.1, NZ_CP017928.1, :NZ_CP018362.1, NZ_CP020358.1 |
| *Klebsiella pneumoniae* | 56-57 | | 10 | NC_016845.1, NC_012731.1, NC_009648.1, NC_011283.1, NC_017540.1, NZ_CP007727.1, NZ_CP008827.1, NC_018522.1, NZ_CP006659.2, NZ_CP011980.1 |
| *Salmonella entérica* | 52 | | 16 | NC_003198.1, NC_003197.2, NZ_CP007270.1, NZ_CP007274.2, NZ_CP007266.2, NZ_CP007362.2, NZ_CP007363.2, NZ_CP007364.2, NZ_CP007289.2, NZ_CP007290.2, NZ_CP007331.2, NZ_CP007344.2, NZ_CP007345.2, NZ_CP007346.2, NZ_CP007352.2, NZ_CP007353.2 |
| *Shigella flexneri* | 51 | | 3 | NC_004337.2, NZ_CM001474.1, NC_004741.1 |
